# Resistance of Common Bean Genotypes to the Broad Mite, *Polyphagotarsonemus latus* (Banks, 1904) (Acari: Tarsonemidae): Offspring Development and Biochemical Basis

**DOI:** 10.3390/insects12100910

**Published:** 2021-10-06

**Authors:** Humberto Godoy Androcioli, Adriano Thibes Hoshino, Maurício Ursi Ventura, Fernando Teruhiko Hata, Marco dos Reis Brugnerotto, Leonel Vinicius Constantino, Francisco de Assis Marques

**Affiliations:** 1 Instituto de Desenvolvimento Rural do Paraná-IAPAR-EMATER (IDR-Paraná), Celso Garcia Cid Highway, km 375, Londrina 86047-902, Brazil; 2 Agronomy Department, Universidade Estadual de Londrina (UEL), Celso Garcia Cid Highway, km 380, Londrina 86057-970, Brazil; adriano.hoshino@uel.br (A.T.H.); mventura@uel.br (M.U.V.); hata@uel.br (F.T.H.); marcoreisbrug@gmail.com (M.d.R.B.); leonel@uel.br (L.V.C.); 3 Chemistry Department, Universidade Federal do Paraná (UFPR), XV de Novembro Street, 1299, Curitiba 80060-000, Brazil; fassismarques@yahoo.com.br

**Keywords:** *Phaseolus vulgaris* L., tolerance, antibiosis, antixenosis, total phenolic compounds, catalase, peroxidase

## Abstract

**Simple Summary:**

The broad mite is a prominent pest, and its management is difficult due to its fast life cycle and farmers’ difficulty in detecting it before the damage is caused. Thus, the use of resistant plants is critical for an integrated pest management program for this mite species. Experiments were conducted to search for common bean varieties with resistance against the broad mite. With our findings, selected genotypes could be used for an integrated pest management program. Carioca Original, one of the most-used varieties in Brazil, had a lower yield, despite low numbers of broad mites. Broad mite populations did not jeopardize the yield of the Verdão and Negrão 11 varieties.

**Abstract:**

The broad mite (BM) *Polyphagotarsonemus latus* is a pest of great prominence for several crops, including the common bean (*Phaseolus vulgaris*). The objective of this study was to select resistant genotypes and to determine chemicals associated with resistance. In the first experiment, BM incidence was assessed for 36 genotypes in a greenhouse study. A group of 10 genotypes was selected according to the development of BM populations. Mite populations and phytometric and biochemical variables were then determined to study eventual differential genotypic responses to mite infestation. Lower numbers of mite mobile forms (larvae + adults) were found on Verdão, Negrão and Carioca Original genotypes. The magnitude of differences reached 5.4 times more BM in the IAC Alvorada than the Verdão genotype. Plant yields were reduced for the genotypes TAA Bola Cheia, IPR Sabiá, IPR Uirapuru, IAC Alvorada and Carioca Original when plants were infested with BM. The yields for LP 13833, BRS Esteio, Negrão 11, Verdão and MD 1133 were similar between infested and non-infested genotypes, indicating tolerance. Verdão and Negrão 11, besides the tolerance, exhibited low offspring development, indicating antibiosis and/or antixenosis. Higher phenolic compound levels were found in the Verdão genotype. Increased contents of catalase and peroxidase were detected for Negrão 11 genotype when infested with BM. This work allowed the detection of common bean genotypes that express resistance and tolerance to BM. These genotypes can be used in places with a history of BM infestation, or used in breeding programs to incorporate these characteristics in other genotypes.

## 1. Introduction

The common bean, *Phaseolus vulgaris* L., is one of the most important leguminous plants. Beans are seeds rich in protein, essential amino acids and bioactive compounds, which are essential for human health [1,2]. The crop is cultivated as a subsistence or cash crop under the most varied edaphoclimatic conditions. Due to the fast vegetative development rate, several crop cycles per year are performed under some tropical and subtropical climates [3].

Crop availability during several months in the field favors arthropod pest development. The broad mite (BM) *Polyphagotarsonemus latus* (Banks, 1904) (Acari: Tarsonemidae) is a polyphagous species which damages nearly 60 plant families around the world [4,5]. BM has been a prominent pest of the common bean for many years [6].

In common bean fields, BM infestations occur initially on few plants. However, under temperatures between 25 and 30 °C, new generations (from egg to adult) may develop weekly, which leads to large populations spreading rapidly in the entire field [7]. Damage caused by *P. latus* infestation in common bean crops may lead to a 50% yield reduction [8].

Due to difficult sampling conditions, preventative strategies using chemical control are generally used. Repeated sprayings can lead to the selection of resistant mite populations, which explains the low efficiency of some acaricides. In addition, the use of broad-spectrum acaricides/insecticides causes the mortality of natural enemies, mostly predatory mites, considerably contributing to the agroecosystems’ imbalance [9].

Host plant resistance is a very desirable strategy for pest management, which is especially advantageous for pests that are difficult to detect, such as the BM. Sometimes, farmers may only realize the pest is present when symptoms appear, and by this time, production is already compromised. Genetic divergence within the same botanical genus might interfere in the biological performance of BMs. Understanding the causes that generate this interference is fundamental to guide breeding programs that promote pest resistance. Several genotypes of “Cambuci’’ pepper (*Capsicum baccatum* L.) were previously screened for resistance to BMs and were used to create the resistant cultivar IAC Ubatuba [10]. Plant resistance has also been proposed for use in BM management on chili (*Capsicum annum* L.) [11,12], citrus (*Citrus aurantium* L. and *Citrus reshni* Hort. ex Tan) [13], cotton (*Gossypium hirsutum* L.) [14] and watermelon (*Citrullus lanatus* spp.) [15].

It is currently suggested that plants perceive herbivore attack through the recognition of substances present in herbivore salivary secretions, which operate as elicitors [16], and by enzymatic reactions resulting from the injury caused by herbivores [17,18,19]. In response to herbivory, the plant quickly starts to produce hydroxyl radicals (HR), superoxide (O^2−^) and hydrogen peroxide (H_2_O_2_) [20,21,22], which induce defense genes and antioxidant enzymes [23].

Peroxidase and catalase are among the many antioxidant enzymes produced by the plant [24] and their concentration fluctuation has been discussed as a first response of the plant towards herbivore insects’ attacks [25,26,27]. Peroxidase is associated with many of the plant’s defense processes in response to biotic and abiotic stresses [23,24,25]. Additionally, the phenylalanine ammonium lyase (PAL) enzyme is associated with lignin biosynthesis, phytoalexins and phenolic compounds, which possess toxic and anti-nutritional properties, participating in the acquired systemic resistance against a wide range of insects [26].

Chemical defense mechanisms are discussed in many plant–mite interactions [27,28,29]. The previously cited compounds take part in biochemical interactions between the infesting mite and host plant, potentially serving as biomarkers [30]. The objective of this study was to select resistant or tolerant common bean genotypes towards BM and determine possible chemical compounds associated with resistance.

## 2. Materials and Methods

The characterization of *P. latus*-resistant genotypes was achieved by setting up two experiments. Initially, screening was carried out to classify the genotypes according to *P. latus* population levels. Next, the relationship between mite incidence, plant yields and plant chemicals in leaf tissues was established.

Plant cultivation and experiments were conducted in a greenhouse at the experimental station of the Instituto de Desenvolvimento Rural do Paraná-IDR-Paraná, Londrina county, PR, Brazil (23°21′22.2′′ S 51°09′51.5′′ W), where the climate was classified as Cfa, according to Köppen, subtropical with hot humid summers. The plants were cultivated in pots (5 L capacity) (one or two plants per pot) containing a mixture of clay soil, sand and cattle manure in a 3:1:1 ratio plus 15 g of 4-14-8 formula (N-P_2_O_5_-K_2_O). The determination of plant chemicals was carried out at the Agricultural Ecophysiology and Biotechnology Laboratory (LEBA) at Universidade Estadual de Londrina (UEL), Londrina, PR, Brazil, and at the Laboratory of Chemical Ecology and Natural Product Synthesis at Universidade Federal do Paraná (UFPR), Curitiba, PR, Brazil.

BM rearing was established from naturally occurring mites on common bean plants and was maintained in a greenhouse. Sweet pepper (*C. annuum*) and common bean plants were used as hosts. Every 20 days, 20 non infested V4-staged plants were introduced into the rearing greenhouse and placed leaning against the older ones to allow the BMs to translocate. The rearing greenhouse was used exclusively for *P. latus* rearing.

### 2.1. Screening Genotypes

The following genotypes maintained in the germplasm bank of IDR-Paraná were selected for the study: ‘Carioca Original’, ‘Verdão’, ‘IAPAR 57’, ‘IPR Curió’, ‘Negrão 11’, ‘MD-1092’, ‘IPR Sabiá’, ‘IAPAR-65’, ‘LP 13-833’, ‘LP 13-827’, ‘IPR Urutau’, ‘IPR Celeiro’, ‘IAPAR 81’, ‘MD-1140’, ‘IPR Gralha’, ‘RAZ-56’, ‘IPR Saracura’, ‘BRS Esteio’, ‘Rio Tibagi’, ‘LP 13-827’, ‘MD-1140’, ‘LP 12-420’, ‘LP 14-55’, ‘IAPAR 139’, ‘RAZ-49’, ‘MD-1133’, ‘LP 11-363’, ‘H-Amarelo’, ‘IPR Corujinha’, ‘RAZ-55’, ‘Capitão’, ‘BRS Pontal’, ‘TAA Bola Cheia’, ‘IPR Tangará’, ‘MD-1092’, and ‘IAC Alvorada’.

A randomized block design with 36 treatments (common bean genotypes) and 4 replicates was used. Each experimental unit consisted of two common bean plants per pot.

When most of the plants had four fully expanded leaves (2 September 2020), approximately 1000 broad mites were released per plant. At 6, 10 and 15 days after infestation (DAI), inspections were conducted on three random leaflets per plant, using a hand magnifying lens (20×) (1 cm^2^) to certify the establishment of the mites.

The BM quantification in the different genotype was carried out 15 and 26 days after infestation. Six pods and eighteen leaflets were collected, six from the upper, six from the medium and six from the lower third of the plants. The leaflets and pods were placed in Petri dishes and kept in polystyrene boxes containing ice at the bottom. Mites were then quantified using a stereoscopic microscope. Quantification was achieved when at least one BM, in mobile form, was found per search. Absence (0 BMs) was recorded just after eight absent observations. According to the values obtained, four frequency classes (%) were assigned: absence of mites on leaflet or pods; from 1 to 5; 6 to 30 and more than 30 mites per cm^2^ of leaflet or pods.

Variance components and predicted genotypic values were estimated using the software Selegen–REML/BLUP [31], by following linear mixed model: y = Xm + Zg + Wp + e.

In this model, ‘y’ is the data vector, ‘m’ is the vector of the effects of the measurement-repeat combinations (assumed to be fixed) added to the overall mean, ‘g’ is the vector of the genotypic effects (assumed to be random), ‘p’ is the vector of permanent environment (random) and ‘e’ is the vector of residuals (random). The capital letters ‘X’, ‘Z’ and ‘W’ represent the incidence matrices for these effects, respectively.

The likelihood ratio test (LRT) was used to verify the significance of the random effects of the statistical model [32], by Chi square test, considering one degree of freedom and a 0.05 probability level.

### 2.2. Resistance of the Genotypes Previously Selected

Ten genotypes of black or colored common bean, which had different BM population densities in the previous experiment, were selected: Negrão 11, Verdão, Carioca Original, BRS Esteio, LP 13-833, IPR-Sabiá, MD- 1133, IPR Uirapuru, TAA Bola Cheia and IAC Alvorada. Plants were grown and experiments conducted in greenhouses (14:10 L:D). The average temperature and relative humidity during the experiments was 25 ± 6 °C and 65 ± 20%, respectively, recorded every half hour by data loggers. Plants were sown on 10 October 2020. Plants from one greenhouse were infested with BMs 40 days after plant emergence, when they had four or five trifoliolate leaves. BM couples (15 per plant) were released in the upper part of the plants. In another greenhouse with similar environmental conditions, the same experiment setup was maintained but plants were not infested.

A randomized block design with 10 treatments (genotypes) and 4 replicates was used. Each plot constituted three pots with two common bean plants; one was used to quantify BM populations, another for plant dry mass and the third for plant yield.

Five consecutive inspections were conducted every five days when BM larvae and adults were counted on nine leaflets per plot (three from high, medium and lower thirds of the plants) using a hand magnifying lens (20×). When no BMs were recorded after five inspections, BM absence was recorded. BMs were also quantified on four pods per plant, observing a 1 cm^2^ portion on one side of the pod. For small pods (<2 cm length), only one portion of 1 cm^2^ was observed; for medium pods (2 to 4 cm length), two portions of 1 cm^2^ each were observed. For large pods (>4 cm length), three portions with 1 cm^2^ each were observed.

Injury degrees caused by BMs were evaluated using the scale proposed by Peña; Bullock, (1994) [5], as follows: 0 = injury absence and leaf expanded; 1 = distal leaf or vicinities with just little silver spots; 2 = shoots deformed or curled leaves; 3 = curled leaves (wrinkled) and silver abaxial surface; and 4 = curled leaves (wrinkled), necrotic apical plant portion, deformed and reduced size of upper leaves and brown leaves. Assessments were carried out every four days, starting immediately before infestation.

Eggs, larvae and adults were determined 15 days after inoculation, when four leaflets of each plant’s third (lower, medium and upper) and all the its pods were collected. The leaflets and pods were placed in acrylic boxes, properly identified and kept in polystyrene boxes containing ice at the bottom. In the laboratory, all the BM forms were quantified using a stereoscopic microscope. This assessment was “destructive” as plants were discarded due to the leaves’ removal.

Dry mass was also estimated at 15 DAI. The whole aerial part of one plant per plot was cut and placed in a paper bag (pods separated from the remaining portion). The material was dried in an air circulation oven, at 60 °C, until constant weight was achieved. The same procedure was adopted for non infested plants.

After plant senescence, the number of total and deformed pods was determined. Seeds were collected and classified using sieves seeds, quantifying those that reached commercial standard.

### 2.3. Quantifying Leaf Chemical Compounds

Leaflets from infested and non infested plants were collected to analyze total phenolic compounds, total soluble proteins, catalase, peroxidase and phenylalanine ammonia lyase. Samples were collected in the morning in the upper third of the plant, one day before plant infestation and 1, 2, 6, 8, 10, 13 and 15 days later. Leaflets were placed in plastic bags and held inside Styrofoam boxes containing ice cubes and transported to the laboratory where the plant material was inspected, removing the mites or dirt particles.

The leaflets to be used for the analysis of total phenolic compounds (TPs) were dried in the oven at 45 °C, until a constant weight was achieved, and crushed. An aliquot of 0.2 g of the crushed material was suspended in 5 mL of 70% ethanol and shaken for 2 h. This material was then filtered with cotton to obtain the ethanolic extract; a 1.0 mL aliquot of this extract was collected and added 1.0 mL of Folin–Ciocalteau 0.90 N and 1.0 mL of 10%sodium carbonate (p/v). This mixture was incubated for 30 min at 25 °C under dark conditions. The mixture’s absorbance was measured at 760 nm in a spectrophotometer (Agilent 8453, Agilent Technologies, Santa Clara, CA, USA) after establishing a standard curve using gallic acid for quantification. Results were expressed as mg of gallic acid equivalent (GAE) per 100 g of dried weight [33].

An additional aliquot of about 0.2 g of fresh leaflets was macerated in a mortar containing 3 mL of buffer solution of potassium phosphate (0.1 M), pH 7.5. The suspension was then placed in a microtube (2.5 mL) and stored in an ultrafreezer (−80 °C). The suspension was centrifuged at 12,000× *g* rpm at 4 °C for 15 min to obtain the supernatant.

For soluble total proteins (STP), to an aliquot of 60 µL of the supernatant, 690 µL of distilled water and 1.5 mL of Bradford reagent (Bio-Rad Protein Assay^®^, Bio-Rad Laboratories, Hercules, CA, USA) diluted in distilled water (1:4) were added. Total protein quantification was achieved using methods described by Bradford [34]. The solution reading was conducted using a spectrophotometer (Agillent 8453, Agilent Technologies, Santa Clara, CA, USA) at 630 nm wavelength, establishing a standard curve.

For catalase (CAT) determination, an aliquot of 100 µL of supernatant was added to 1.9 mL of buffer solution of 0.05 M potassium phosphate, pH 7.0, containing 12.5 mM hydrogen peroxide. The catalase enzymatic activity was determined by absorbance at 240 nm wavelength, using the extinction molar coefficient of 36 M^−1^ cm^−1^ [35]. After establishing the standard curve, the contents of CAR were determined (mmol·H_2_O_2_/mg protein).

For peroxidase (POX), an aliquot of 100 µL of supernatant was added to 1.8 mL of the solution containing 250 µL of guaiacol + 306 µL of peroxide of hydrogen. The total volume was completed for 100 mL using a buffer solution of 0.01 M potassium phosphate at pH 6.0. Absorbance was then determined at 470 nm. Peroxidase activity was determined by the conversion of guaiacol to tetraguayacol [36]. After determination of the standard curve, POX contents were determined (UAbs/min.mg protein).

For phenylalanine ammonia lyase (PAL) determinations, an aliquot of 40 µL was added to 1 mL of Tris-HCl^®^ (Invitrogen, Waltham, EUA), pH 8.0 and mixed in a vortex for 5 s. Next, an aliquot of 0.75 mL was transferred to the microtube; 0.5 mL of Tris-HCl^®^ was added, pH 8.0, and 0.25 mL of phenylalanine solution (49.6 mg mL^−1^) was mixed in a vortex for 10 s. Colorimetry of the trans-cinnamic acid was accomplished in the phenylalanine substrate, using a procedure in accordance with Kuhn (2007) [37]. The obtained solution was then placed in a water bath for 50 min at 40 °C. Next, the reaction was interrupted by placing the tubes in an ice bath for one minute and reading at 290 nm, using a quartz bucket. After determining the standard curve, levels of PAL were determined (UAbs/min.mg protein).

A large variation in the quantity of foliar compounds was identified as time passed for the same genotype of common bean, with or without BM infestation (Figures S1–S5). Thus, the best means of identifying each quantity of foliar compounds, for a determined genotype, considering the eight evaluations performed, was through the integration of the area under the curve (AUC). The AUC was estimated using the AgroR package [38].

### 2.4. Statistics

Data were submitted to homoscedasticity and normality tests to verify the assumptions of the parametric analysis. When assumptions were not met, the data were transformed using the square root (x + 0.5).

The Tukey test was used, in split-plot design, to compare the number of mobile forms of BMs (larvae + adults) among genotypes and thirds of plants.

The Scott–Knott test was used to compare the variable number of BM 15 DAI and the AUC of the analyzed compounds. The t test was accomplished within each genotype to compare infested and non-infested plants for the variables: AUC of the foliar compounds; dry mass of aerial portion of the plants; percent of curl pods, and number and weight of commercial seeds per plant. Spearman correlation was performed between the variable number of mites determined by destructive assessments using stereoscopic microscopy and the number of mites determined by a hand magnifying lens at 15 DAI vs. leaf biochemical compounds.

Leaf biochemical compounds, genotypes, infested or non-infested plants and BMs were submitted to an analysis of principal components (APC) to study their associations.

The AgroR package was used for statistical analysis [38].

## 3. Results and Discussion

**Figure 1:**
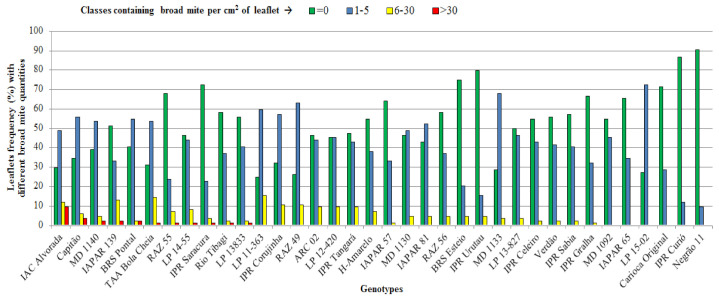
Frequency distribution of four classes of frequencies of broad mite in 1 cm^2^ of leaflets in different common bean genotypes. Londrina-PR, 2020.

In the screening tests, genotypes IAC Alvorada, Capitão, MD1140, IAPAR 139, BRS Pontal, TAA Bola Cheia, RAZ 55, LP 14-55, IPR Saracura, Rio Tibagi and LP 13,833 had the largest number of BM offspring on their leaflets, while the genotypes MD 1092, IAPAR 65, Carioca Original, IPR Curió and Negrão 11 had the lowest number of BM offspring on their leaflets (Figure 1).

Through the *likelihood-ratio test*—LRT, using deviance analysis, a significant effect (*p* < 0.05) of the genotypes on the number of BMs was detected for the whole plant mite count (Table 1). When each third of the plant was analyzed separately, no difference was detected (*p* > 0.05).

Regardless of the method with which the BM quantification was performed, in thirds or with the sum of these, low magnitude values (0.07) of the heritability estimate to total genotypic effects were observed. The use of two evaluations meant that an average magnitude (0.38) of genotypes’ heritability (*h^2^_mg_*) was reached, as a consequence of the low (0.08) estimated repeatability coefficient (*ρ*), which indicates the need to increase the number of evaluations to improve the precision in selecting which genotypes provide the greatest or smallest BM population (Table 2). The values were classified according to the criteria previously suggested [39].

The genotypic values allowed the classification of the genotypes according to suitability for growth of the BM offspring. In general, under a suitable temperature (25 to 28 °C) and humidity (65 to 90%), a new generation of BMs was accomplished in just one week, with the adult longevity lasting less than two weeks [7,40,41]. Hence, the population assessed may be considered “offspring” of the mites used for infestation. For example, in IAC Alvorada, the genetic effects (g), genotypic values (GV) and phenotypic mean (f) exhibited the highest values and in Carioca Original, the lowest (Table 3). Considering the genetic and phenotypic values, genotypes that provided different BM population growths were chosen to conduct the following experiment.

**Figure 2:**
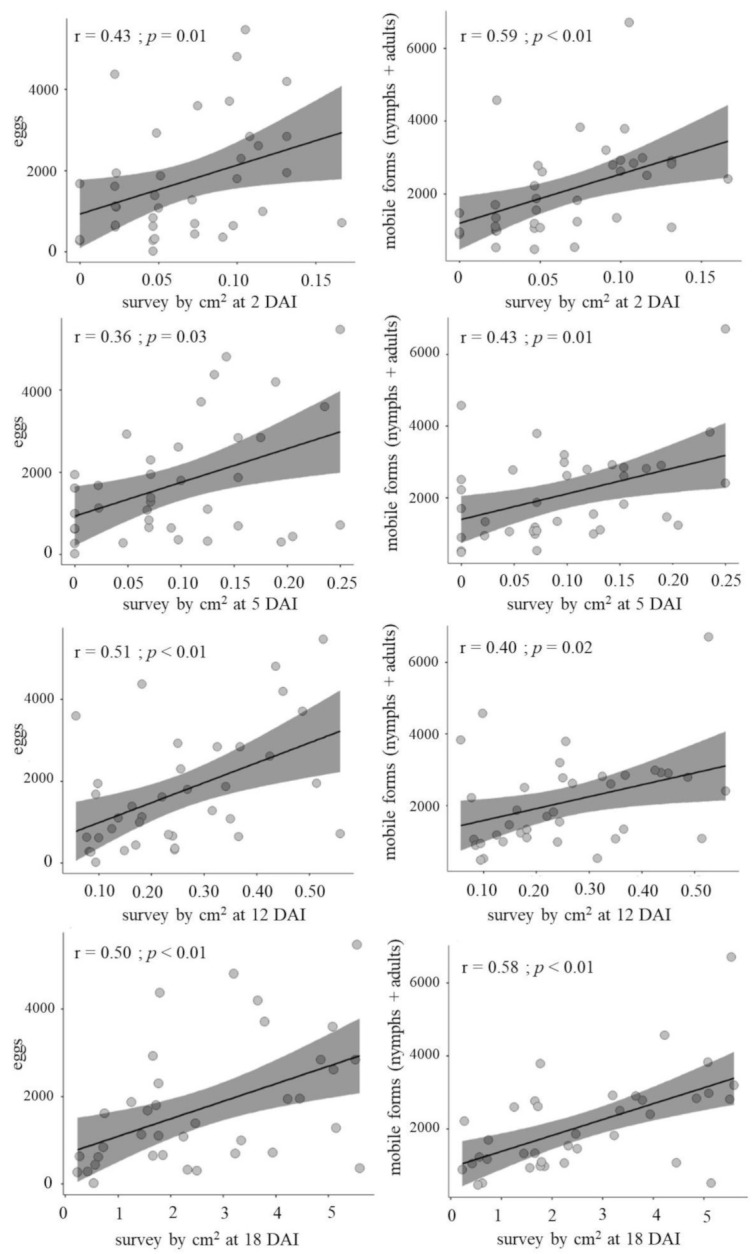
Spearman coefficient (r) of the number of broad mites (*Polyphagotarsonemus latus*) on common bean leaves 15 days after infestation assessed through destructive assessment vs. number of mobile forms assessed using hand magnifying lenses. *p*-Values indicate correlation significance. Londrina-PR, Brazil, 2021.

A positive correlation between the number of mites assessed by a hand magnifying glass (20×) at 2, 5, 12 or 18 DAI and destructive assessment (15 DAI) was obtained (*p*-value ≤ 0.03) (Figure 2). Although high correlation values were not verified (among 0.36 and 0.59), these findings suggest that it is possible to estimate the BM population growth using a hand magnifying glass over a 1 cm^2^ area.

**Figure 3:**
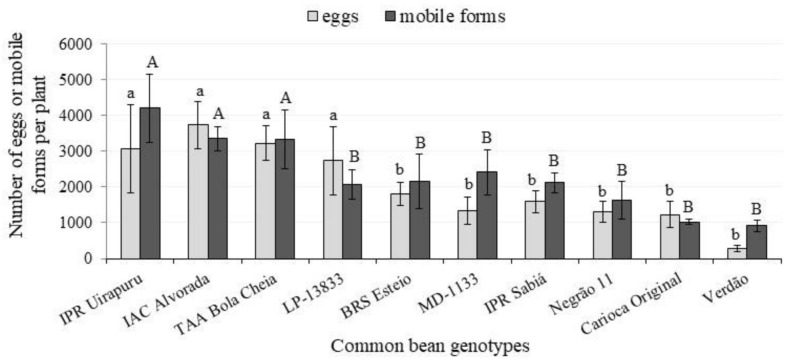
Number of eggs (clear column) and mobile forms (larvae + adults) (dark column) of *Polyphagotarsonemus latus* per plant 15 days after infestation of common bean genotypes. Bars over the columns indicate standard errors. Columns with the same letter, lower case (a, b) for “eggs” and capital letters (A, B) for “mobile forms”, do not differ by Scott–Knott test (α = 5%).

In the second experiment, the number of BMs varied among genotypes (F = 3.993 and 3.223 *p* < 0.01) and plant thirds (F = 27.4209 and 40.713; *p* < 0.01) for eggs and mobile forms, respectively. Overall, higher BM populations were recorded in the upper third of the plant, mostly for the most susceptible genotypes (Table 4). This is in accordance with the previous report that broad mites were mostly found to feed on younger leaves [42]. As in the previous experiment, Verdão, Negrão and Carioca Original genotypes had the lowest numbers of mite mobile forms, mostly in the medium and upper thirds. ‘Verdão’ also hosted the lowest numbers of eggs for both the upper and medium thirds (Table 4). The number of BM mobile forms in IAC Alvorada was 5.4 times higher than for the Verdão genotype (Table 4, Figure 3).

**Figure 4:**
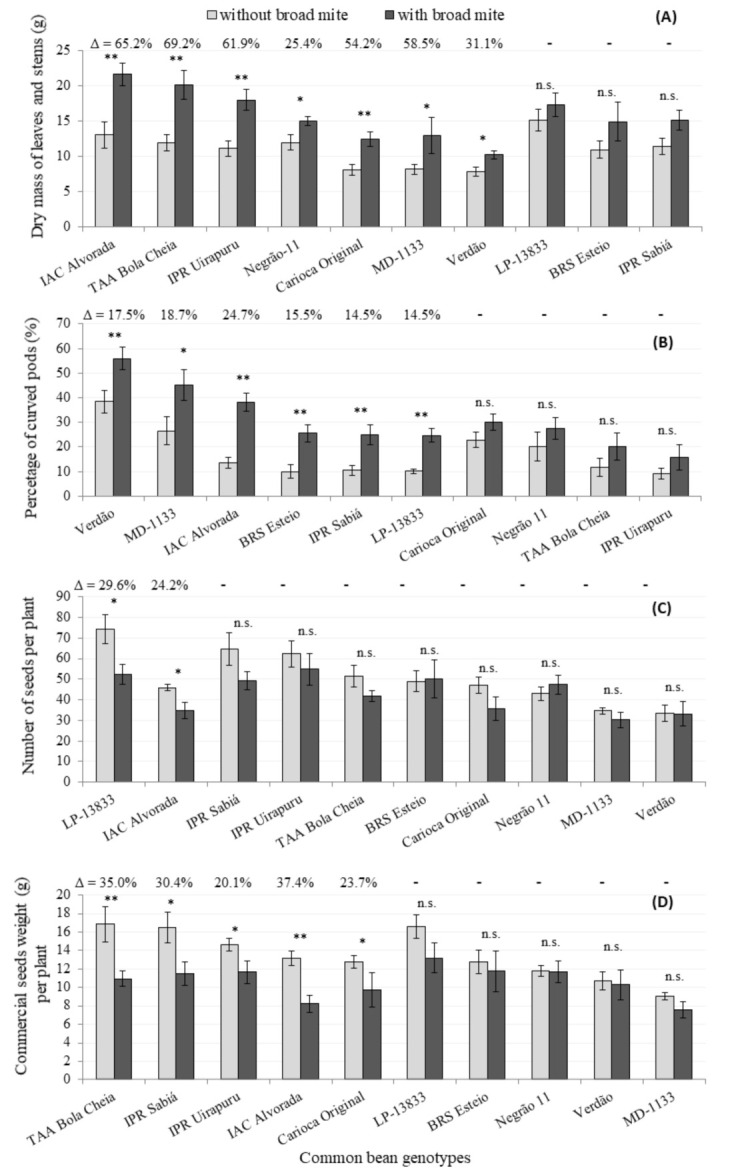
Dry mass of the leaves and stems (**A**), percent of curved pods (**B**), number of commercial seeds (**C**) and commercial weight of seeds per plant (**D**) of genotypes of common bean infested (dark column) and non infested (clear column) with *Polyphagotarsonemus latus*. Single (*) and double (**) asterisks over bars indicate significant at 5% and 1%, respectively, by Student *t* test, while n.s. indicates not significant. Δ values indicate the increment when infested vs. non infested plants were compared.

Overall, a larger amount dry mass of aerial parts on BM-infested plants than non-infested plants was found, except for in LP 13833, BRS Esteio and IPR Sabiá genotypes (Figure 4). The increase in the dry mass varied from 25.4% for Negrão 11 to 69.2% for TAA Bola Cheia. A moderate and positive correlation was verified between the number of mobile forms and dry mass (r = 0.67; *p*-value = 0.03). Despite the BM occurrence on plants, no leaf shed was observed. Herbivory causes a series of changes in plant metabolisms that occur in order to isolate the affected tissue to limit further damage on the whole plant [43]. Possibly, BM feeding triggered higher suberization in the damaged tissues, inducing a larger amount of dry biomass.

Plants with BM had curled and stiff leaves as a consequence of tissue suberization without an apparent reduction in leaf area, probably due to the short period between infestation and dry mass determination (15 days). Probably, a different result would occur if the dry mass determination were performed close to the harvest period, al-lowing enough time to verify reduction in leaf area or plant size. Another study found that the BM occurrence did not reduce the dry mass of sweet pepper plants [44]. Thus, is important to consider the time elapsed between BM infestation and dry mass deter-mination.

The BM infestation led to higher percent of curved pods for six genotypes. The percent of curved pods ranged from 14.5 (IPR Sabiá and LP-13833) to 24.7% (IAC Alvorada) (Figure 4). The property of curved pods has an important effect, mostly for materials that are freshly commercialized, because this trait depreciates the final product. No correlation between the number of curved pods and the number of mobile BMs was observed (r = −0.26; *p*-value = 0.48), which indicates a different sensibility of the genotypes regarding pod deformation due to BM occurrence. For the Verdão genotype, a higher increment of curved pod (17.5%) was observed, despite hosting a relatively lower BM population.

Regarding the number of seeds per plant, most genotypes were not affected by the number of BM mobile forms. For LP 13,833 and IAC Alvorada, the number of seeds was reduced (29.6 and 24.2%, respectively) (Figure 4). The number of seeds per plant also did not correlate with the number of mobile forms (r = −0.09; *p*-value = 0.82).

Plant yields were reduced for the genotypes TAA Bola Cheia, IPR Sabiá, IPR Uirapuru, IAC Alvorada and Carioca Original, when plants were infested with BM. Decreases ranged from 20.1 to 37.4%, which was lower than previous reports of 50% yield reduction in other genotypes of common bean [7]. The yields for LP 13833, BRS Esteio, Negrão 11, Verdão and MD 1133 were similar between infested and non-infested plants (Figure 4), which may suggest these genotypes as an eventual source of tolerance to the BM.

For the Carioca Original genotype, although a relatively lower number of BM offspring than IPR Uirapuru, IAC Alvorada and TAA Bola Cheia was recorded (Table 4 and Figure 3), yield reduction was significant (Figure 4). The same was not true for Verdão and Negrão 11 (Table 4 and Figure 4). This suggests that the latter two genotypes provide negative effects on the pest through antibiosis and or antixenosis, and also exhibit tolerance towards the BM populations.

For the genotypes LP 13833, MD 1133 and BRS Esteio, on which an intermediate number of BMs was recorded (Table 4), yields were similar between BM-infested and non-infested plants, which may suggest plant tolerance. The same was not found for the IPR Sabiá genotype, which showed significant yield reduction.

For the genotypes with the highest numbers of BMs, IAC Alvorada, TAA Bola Cheia and IPR Uirapuru (>3000), significant yield losses were observed (Figure 4).

As also determined for the number of seeds per plant, no correlation between BM numbers and the weight of seeds per plant was found (r = 0.47; *p*-value = 0.17), probably due to the tolerance mechanisms exhibited by some genotypes.

**Figure 5:**
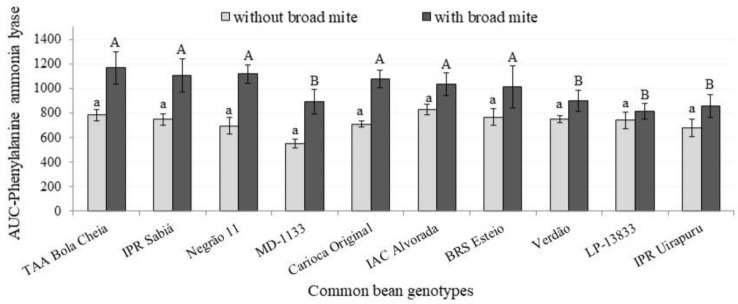
Area under the curve of phenylalanine ammonia lyase content in leaves from different common bean genotypes infested (dark column) and not infested (clear column) with *Polyphagotarsonemus latus*. Values obtained from assessments one day before and 1, 2, 6, 8, 10, 13 and 15 days after *P. latus* infestation. Columns with the same letter, lower case (a, b) for “without broad mite” and capital letters (A, B) for “with broad mite”, do not differ by Scott–Knott test (α = 5%).

The calculated AUC of phenylalanine ammonia lyase content was not affected by the interaction between mite infestation and genotype (*p* = 0.07), and this enzyme was only affected by the genotype (*p* < 0.01). A higher AUC for phenylalanine ammonia lyase content was found in Negrão 11, BRS Esteio, Carioca Original, IPR Sabiá, TAA Bola Cheia and IAC Alvorada (Figure 5).

**Figure 6:**
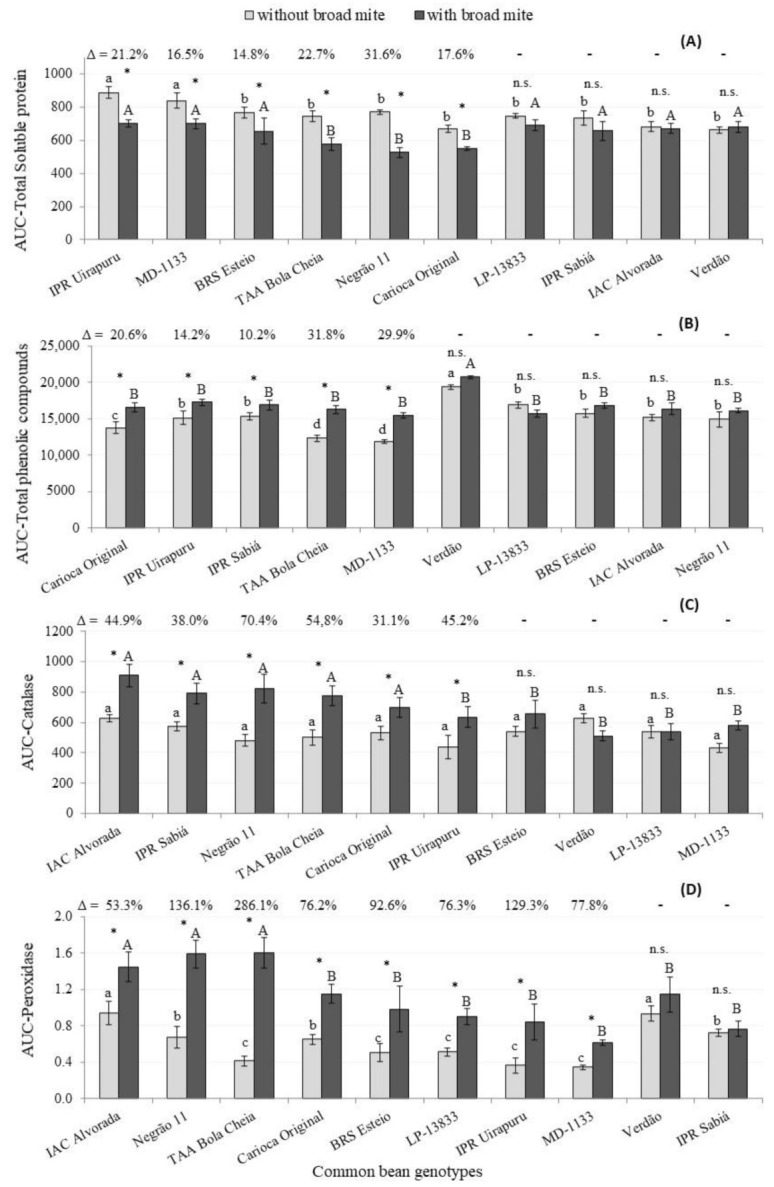
Areas under the curve total soluble proteins (**A**), total phenolics (**B**), catalase (**C**) and peroxidase (**D**) contents in leaves from different common bean genotypes infested (dark column) and not infested (clear column) with *Polyphagotarsonemus latus*. Values obtained from assessments one day before and 1, 2, 6, 8, 10, 13 and 15 days after *P. latus* infestation. Columns with the same letter, lower case (a, b, c) for “without broad mite” and capital letters (A, B) for “with broad mite”, do not differ by Scott–Knott test (α = 5%). Asterisks over bars indicate significant difference by Student’s *t* test (*p* < 0.05) and n.s. indicates not significant. Δ values indicate the increment when infested vs. non-infested plants were compared.

The calculated AUC of the total proteins from BM-infested plants of the genotypes IPR Uirapuru, MD 1133, TAA Bola Cheia, Negrão 11, BRS Esteio and Carioca Original were lower than those of the non-infested ones (reduction from 14.8% to 31.5%) (Figure 6A). Another study found similar results, in which common beans, infested with thrips (*Frankliniella occidentalis* Pergande), produced less soluble proteins compared to non-infested plants [45]. In general, diminishing protein contents indicates an allocation of photoassimilates for defensive proposals [46].

However, the BM-infested plants of the genotypes Carioca Original, IPR Sabiá, IPR Uirapuru, TAA Bola Cheia and MD 1133 had higher values of AUC contents of phenolic compounds than non-infested plants, with increases from 14.2% to 31.8% (Figure 6B). These changes in the contents are due to rapid upregulation in the phenylpropanoid pathway to provide phenolic production and accumulation under stress [47]. Phenolic compounds influenced by fertilization were also suggested in the diminishing populations of the spider mite *T. urticae* in strawberries [48]. In this study, the highest contents were found for the Verdão genotype, whether infested or not. In this genotype, lower numbers of eggs and mobile forms were also found (Table 4 and Figure 3) and the mite populations had no effect on yield. These results suggest an eventual importance of these compounds on resistance expression.

Peroxidase and catalase reduce the reactive oxygen species (ROS) accumulation and their activities indicate whether biotic or abiotic stress has been induced [49]. In the present study, increasing values for the foliar catalase contents of IAC Alvorada, IPR Sabiá, Negrão 11, TAA Bola Cheia, Carioca Original and IPR Uirapuru genotypes were observed when plants were infested. These increments ranged from 31.1% to 70.4% Differences in the catalase quantity between genotypes was found only when the plants were infested; similar values were observed for non-infested ones (Figure 6C).

BM infestation on IAC Alvorada, Negrão 11, TAA Bola Cheia, Carioca Original and led to a higher AUC for the peroxidase leaf contents (Figure 6D). These increments were notably high, from 53.3 (IAC Alvorada) to 286.1% (TAA Bola Cheia). Higher levels of catalase and peroxidase were observed in common bean plants infested with *F. occidentalis* [45].

Catalase and peroxidase enzymes are generally produced by stressed plants. Previous studies characterized higher catalase production together with dismutase enzymes leading to cassava plants’ resistance to *Tetranychus cinnabarinus* (Boisduval) (Acari: Tetranychidae) [50]. The catalase and peroxidase production was also enhanced in response to the whitefly *Bemisia tabaci* (Gennadius) feeding [51]. The authors suggested activities of the enzymes may contribute to the bioprotection of black gram plants (*Vigna mungo* L. Hepper) against *B. tabaci* infestation. Previously, biochemical traits were also associated with BM development on *Cochorus alitorius* L. and *Corchorus capsularis* L. genotypes [52]. The herbivory behavior of an arthropod may not always lead to a joint increase in catalase and peroxidase enzymes; one study indicates that the presence of *T. urticae* in soy (*Glycine max* L. (Merril)) resulted in higher levels of peroxidase, but had no effect on catalase levels [53].

No correlation was found between chemical compounds (total phenolic compounds, total soluble proteins, catalase, peroxidase and phenylalanine ammonia lyase) and the number of eggs (r: 0.03 to 0.27; *p*-value: 0.45 to 0.95) or mobile forms (r: 0.09 to 0.45; *p*-value: 0.19 to 0.81) in the assessment using 15 DAI (Table 5). In previous studies, positive correlation was established between BM susceptibility and protein/nitrogen contents [54,55] and negative correlation with total phenolic compounds in chili cultivars [54]. Catalase and phenolic compounds were also associated with BM infestation in chili in other studies [56].

**Figure 7:**
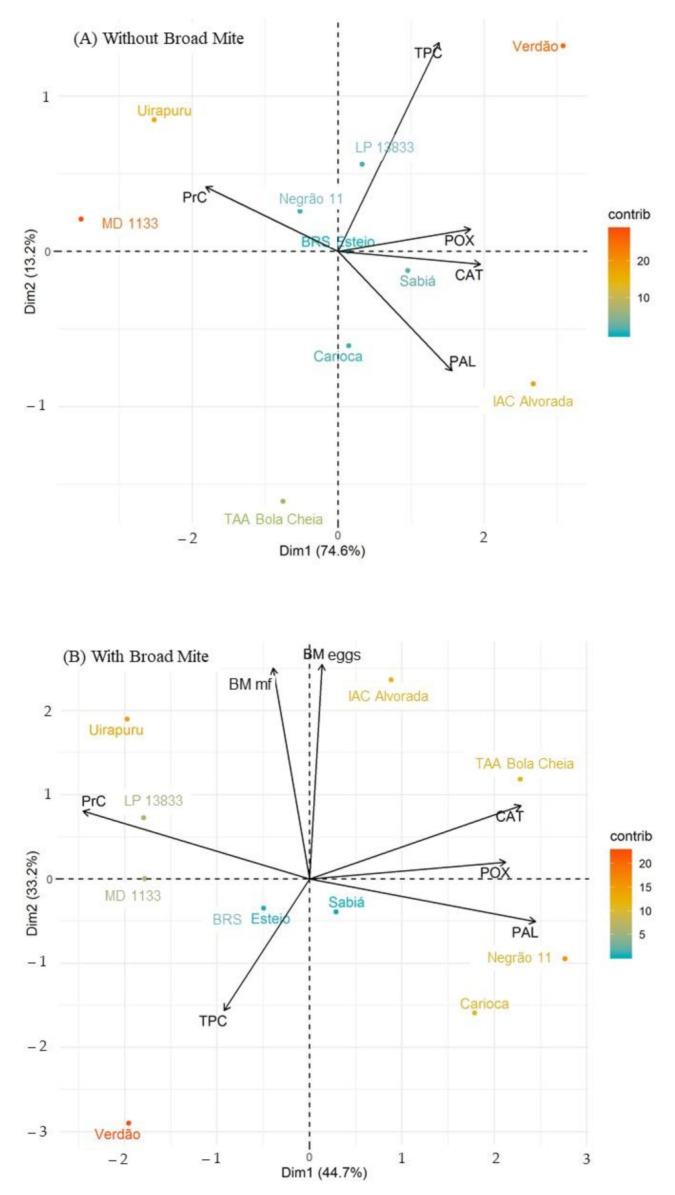
Analysis of principal components between the leaf contents of proteins (PrC), total phenolic compounds (TPC), catalase (CAT), peroxidase (POX), phenylalanine ammonia lyase (PAL), number of eggs of the broad mite *Polyphagotarsonemus latus* (BM eggs) and mobile forms (BM-mf) indicated by the eigenvector and common bean genotypes indicated by the eigenvalues in non-infested (**A**) and infested (**B**) with *P. latus*. Londrina-PR, 2021.

The analyses of the principal component suggest the relationship of the leaf contents of the analyzed compounds of BM infested and non-infested plants. The sum of components explains 87.8 and 77.9% of the variables, respectively. Independent of infestation, Verdão genotype plants were associated with total phenolic compounds, while Uirapuru and MD 1133 genotype plants were associated with proteins. The LP 13,833 genotype was related to protein contents when plants were infested with BMs. The IAC Alvorada genotype was related to phenylalanine ammonia lyase when plants were not infested with BMs. Infested Negrão 11 and Carioca Original genotype plants were related with phenylalanine ammonia lyase. The TAA Bola Cheia genotype was only related to catalase when plants were infested. The genotype most associated with eggs and mobile forms was IAC Alvorada (Figure 7).

In summary, a similar weight of seeds for BM-infested and non-infested plants was observed for the genotypes LP 13833, Negrão 11, Verdão, BRS Esteio and MD 1133, indicating plant tolerance. Verdão and Negrão 11, besides the tolerance, exhibited low offspring development, indicating antibiosis and/or antixenosis. Higher phenolic compounds levels were measured for the Verdão genotype. Higher levels of catalase and peroxidase were observed for the Negrão 11 genotype when the plants were infested with BMs. These genotypes could serve as sources of resistance in common bean resistance to BMs in plant breeding programs.

## Figures and Tables

**Table 1:** Analyses of deviance (ANADEV): deviance (Dev) and likelihood-ratio test (LRT) for genotypes’ effects and complete model for quantification of *Polyphagotarsonemus latus* in 36 common bean genotypes in the three thirds of the plant: higher (HT), medium (MT) and lower (LT) and sum of the whole plant (WP).

Effect	HT		MT		LT		WP	
	Dev	LRT	Dev	LRT	Dev	LRT	Dev	LRT
Genotype	2164.70	2.67 ^ns^	1547.70	1.28 ^ns^	1341.44	0.01 ^ns^	2281.66	4.05 *
Complete model	2162.03		1546.42		1341.43		2277.61	

n.s. = not significant, * = significant at 5% by Chi square test.

**Table 2:** Variance components and genetic and non-genetic parameters for *Polyphagotarsonemus latus* quantification in 36 common bean genotypes in three plant thirds: higher (HT), medium (MT) and lower (LT) and sum of the whole plant (WP).

Components/Parameters	HT	MT	LT	WP
*σ^2^_g_*	51.37	4.35	0.07	100.73
*σ^2^_perm_*	7.78	7.23	0.55	12.94
*σ^2^_e_*	836.01	83.23	43.74	1258.53
*σ^2^_f_*	895.15	94.81	44.36	1372.20
*h^2^_g_*	0.06	0.05	0.01	0.07
*h^2^_mg_*	0.32	0.26	0.01	0.38
*ρ*	0.07	0.12	0.01	0.08
*c^2^_perm_*	0.01	0.08	0.01	0.01
Overall means	18.03	8.43	5.11	31.57

*σ^2^_g_*: genetic variance; *σ^2^_perm_*: permanent environmental variance; *σ^2^_e_*: residual variance; *σ^2^_f_*: phenotypic variance; *h^2^_g_*: total genetic effect heritability; *h^2^_mg_*: overall genetic mean heritability; *ρ*: coefficient of repeatability; *c^2^_perm_*: coefficient of determination of permanent environment.

**Table 3:** Genetic effects (g), genotypic values (GV) and phenotypic mean (f) for common bean genotypes assessed for the number of *Polyphagotarsonemus latus* on the whole plant.

Genotypes	g	GV	f	Genotypes	g	GV	f
Carioca Original	−8.08	23.49	12.00	Rio Tibagi	−1.12	30.45	31.57
Verdão	−6.82	24.76	15.57	LP 13-827	−0.99	30.58	29.00
IAPAR 57	−5.95	25.62	16.13	MD-1140	0.36	31.93	32.50
IPR Curió	−5.81	25.76	16.50	LP 12-420	0.50	32.07	32.88
Negrão 11	−5.67	25.91	16.88	LP 14-55	0.89	32.46	33.88
MD-1092	−4.12	27.45	20.88	IAPAR 139	1.08	32.65	34.38
IPR Sabiá	−4.08	27.50	21.00	RAZ-49	1.18	32.75	34.63
IAPAR-65	−3.79	27.78	21.75	MD-1133	1.37	32.94	35.13
LP 13-833	−3.64	27.93	22.13	LP 11-363	1.62	33.19	39.36
LP 13-827	−3.50	28.07	22.50	H-Amarelo	2.19	33.76	37.25
IPR Urutau	−3.02	28.56	23.75	IPR Corujinha	2.86	34.44	39.00
IPR Celeiro	−2.87	28.7	24.13	RAZ-55	3.06	34.63	39.50
IAPAR 81	−2.58	28.99	24.88	Capitão	3.39	34.97	40.38
MD-1140	−2.40	29.17	28.00	BRS Pontal	3.59	35.16	40.88
IPR Gralha	−2.15	29.42	26.00	TAA Bola Cheia	4.84	36.41	44.13
RAZ-56	−1.86	29.71	26.75	IPR Tangará	7.73	39.30	51.63
IPR Saracura	−1.75	29.82	29.86	MD-1092	9.08	40.65	55.13
BRS Esteio	−1.62	29.95	27.38	IAC Alvorada	28.07	59.64	104.38

**Table 4:** Number of eggs and mobile forms (larvae + adults) (*n* = 4) of *Polyphagotarsonemus latus* on leaves and pods in the higher, medium and lower third of plants of common bean (*Phaseolus vulgaris*) genotypes, 15 days after infestation. Londrina-PR, 2021.

Genotypes	Higher			Medium			Lower		
	Eggs								
BRS Esteio	1284.8	AB	a	256.8	AB	b	264.3	A	b
Carioca Original	453.8	BC	a	391.5	AB	a	381.5	A	a
IAC-Alvorada	2368.0	A	a	796.5	AB	b	571.8	A	b
LP 13833	1286.7	AB	a	381.0	AB	b	160.8	A	b
MD 1133	772.0	ABC	a	303.3	AB	a	261.8	A	a
Negrão 11	464.3	BC	a	681.5	AB	a	163.0	A	a
IPR Sabiá	748.5	ABC	a	658.3	AB	ab	185.3	A	b
TAA Bola Cheia	1532.3	AB	a	1075.3	A	ab	621.3	A	b
IPR Uirapuru	1590.5	AB	a	834.0	AB	ab	651.3	A	b
Verdão	78.5	C	a	111.8	B	a	77.5	A	a
**Genotypes**	**Mobile Forms (Larvae and Adults)**								
BRS Esteio	931.3	ABC	a	735.3	AB	a	487.8	A	a
Carioca Original	404.5	C	a	315.5	B	a	299.3	A	a
IAC-Alvorada	2078.0	A	a	875.8	AB	b	407.8	A	b
LP 13833	1223.8	ABC	a	462.0	AB	b	378.8	A	b
MD 1133	1047.5	ABC	a	816.3	AB	a	551.3	A	a
Negrão 11	535.5	BC	a	773.5	AB	a	311.8	A	a
Sabiá	1233.8	ABC	a	643.8	AB	ab	251.0	A	b
TAA Bola Cheia	1758.5	AB	a	859.5	AB	b	716.0	A	b
Uirapuru	1861.5	A	a	1439.8	A	ab	900.0	A	b
Verdão	383.0	C	a	219.3	B	a	314.5	A	a

Means followed by the same letter, capital letter in the column and lower case in the line did not differ by the Tukey test (α = 5%).

**Table 5:** Spearman coefficient (r) between egg numbers, larvae (La) + adults (Ad) of *Polyphagotarsonemus latus* at 15 days after infestation and the area under the curve (AUC) for the leaf protein, total phenolic compounds (TPC), catalase (Cat), peroxidase (Pox) and phenylalanine ammonia lyase (PAL). Londrina-PR, 2021.

Correlation	AUC-Protein		AUC-TPC		AUC-Cat		AUC-Pox		AUC-PAL	
	Eggs	La + Ad	Eggs	La + Ad	Eggs	La + Ad	Eggs	La + Ad	Eggs	La + Ad
r	0.27	0.45	0.07	0.22	0.18	0.09	0.03	0.19	0.22	0.35
*p*-value	0.45	0.19	0.86	0.54	0.63	0.81	0.95	0.61	0.54	0.33

## Data Availability

The data presented in this study are available in article.

## Supplementary Materials

The following are available online at https://www.mdpi.com/article/10.3390/insects12100910/s1. Figure S1: Total protein content in different common bean genotypes with (dark line) and without (grey line) broad mite—BM (*Polyphagotarsonemus latus*) infestation. Numbers in the *x*-axis refers at days before infestation (D.B.I,) or days after infestation (D.A.I). Londrina-PR, 2020. Figure S2: Total phenolic compounds content in different common bean genotypes with (dark line) and without (grey line) broad mite—BM (*Polyphagotarsonemus latus*) infestation. Numbers in the *x*-axis refers at days before infestation (D.B.I,) or days after infestation (D.A.I). Londrina-PR, 2020. Figure S3: Catalase content in different common bean genotypes with (dark line) and without (grey line) broad mite—BM (*Polyphagotarsonemus latus*) infestation. Numbers in the *x*-axis refers at days before infestation (D.B.I,) or days after infestation (D.A.I). Londrina-PR, 2020. Figure S4: Peroxidase content in different common bean genotypes with (dark line) and without (grey line) broad mite—BM (*Polyphagotarsonemus latus*) infestation. Numbers in the *x*-axis refers at days before infestation (D.B.I,) or days after infestation (D.A.I). Londrina-PR, 2020. Figure S5: Phenylalanine ammonia lyase content in different common bean genotypes with (dark line) and without (grey line) broad mite—BM (*Polyphagotarsonemus latus)* infestation. Numbers in the *x*-axis refers at days before in-festation (D.B.I,) or days after infestation (D.A.I). Londrina-PR, 2020.
