# Supplementary material for: Resistance of Common Bean Genotypes to the Broad Mite, Polyphagotarsonemus latus (Banks, 1904) (Acari: Tarsonemidae): Offspring Development and Biochemical Basis
Source: Insects. 2021 Oct 6;12(10):910. doi: 10.3390/insects12100910 (PMC8540688; doi:10.3390/insects12100910)
Supplement: Supplementary file 1 [file insects-12-00910-s001.zip › insects-1317373-supplementary.pdf]

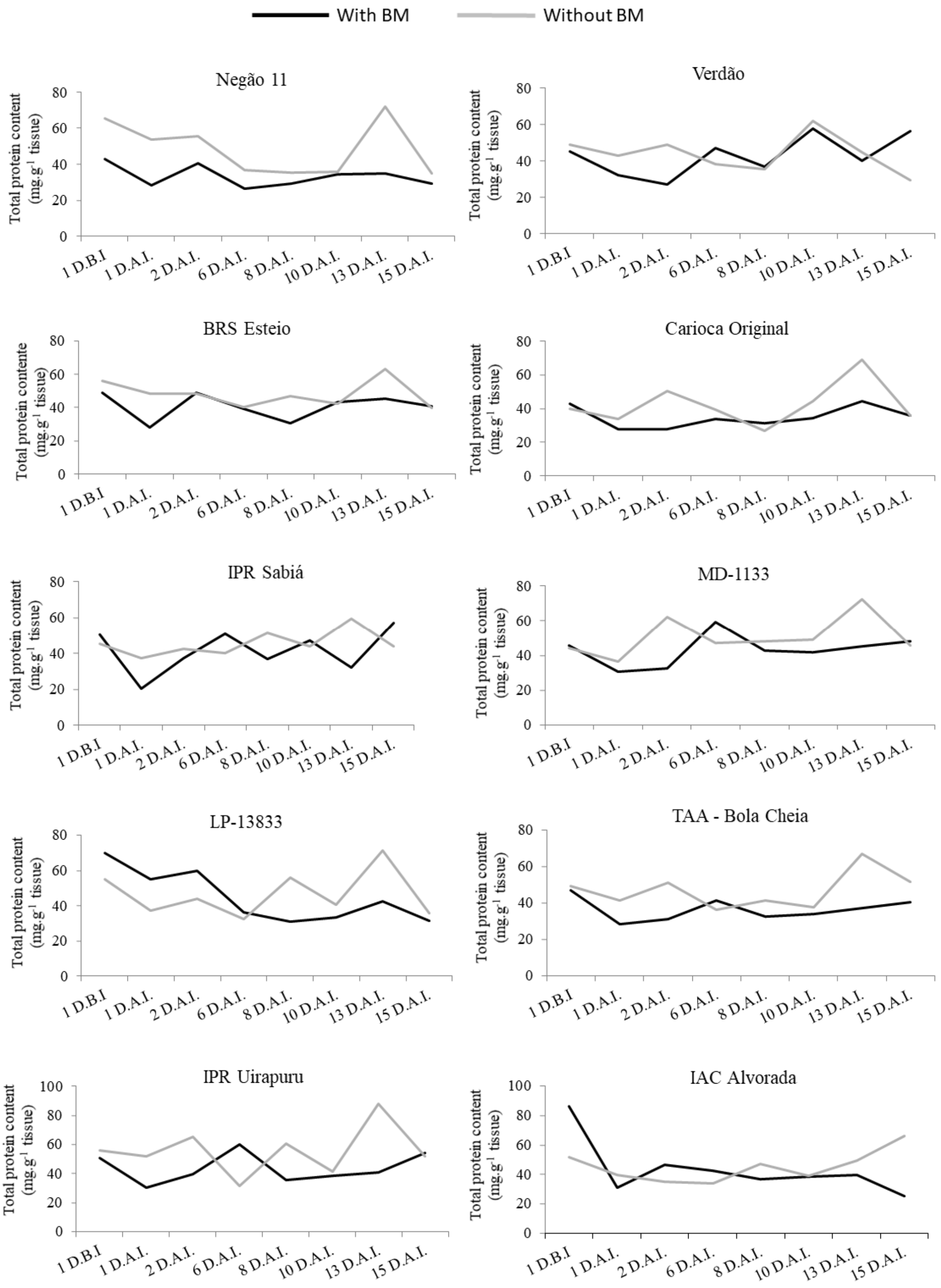

Figure S1: Total protein content in different common bean genotypes with (dark line) and without (grey line) broad mite - BM (*Polyphagotarsonemus latus*) infestation. Numbers in the x-axis refers at days before infestation (D.B.I,) or days after infestation (D.A.I). Londrina-PR, 2020.

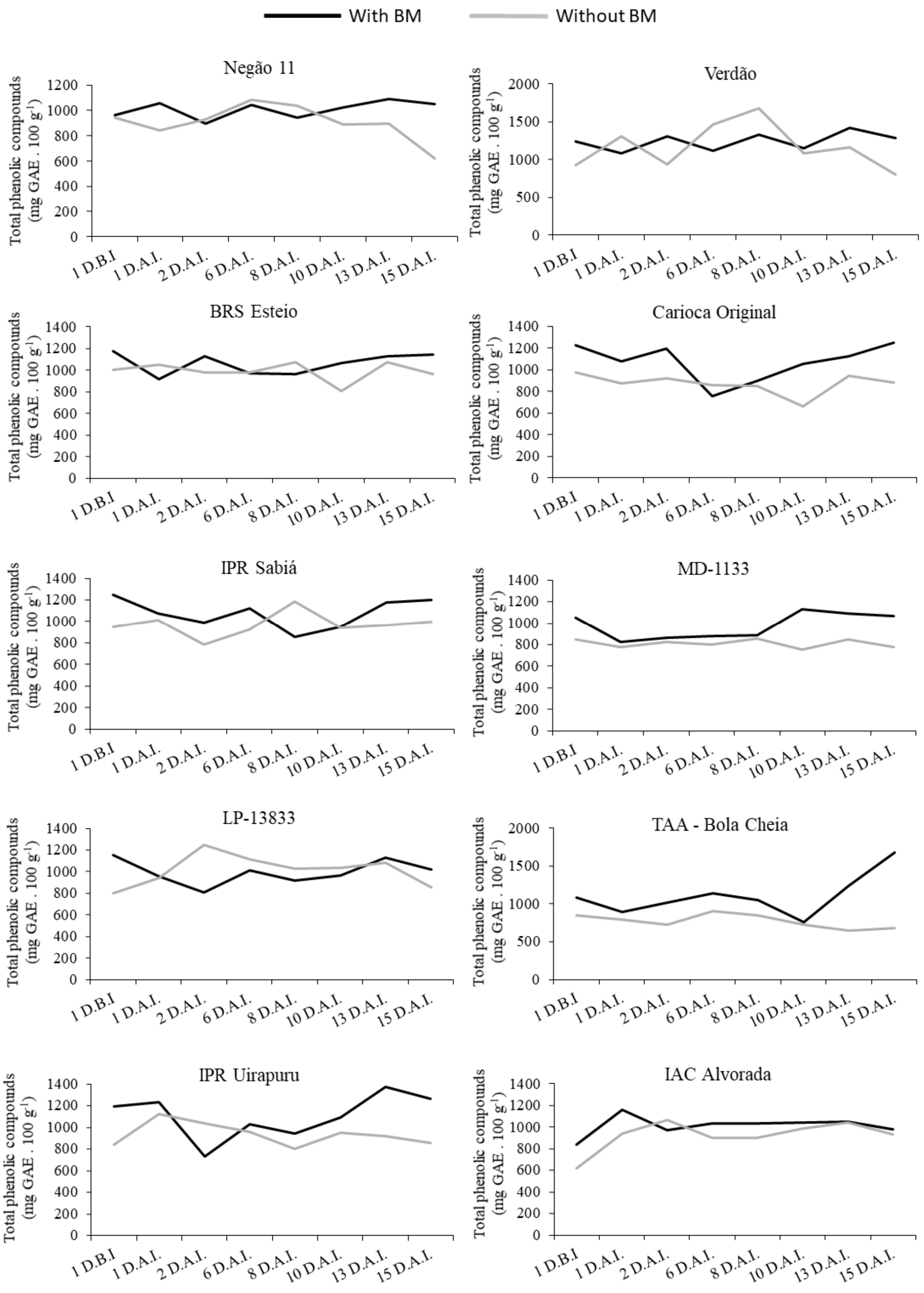

Figure S2: Total phenolic compounds content in different common bean genotypes with (dark line) and without (grey line) broad mite - BM (*Polyphagotarsonemus latus*) infestation. Numbers in the x-axis refers at days before infestation (D.B.I,) or days after infestation (D.A.I). Londrina-PR, 2020.

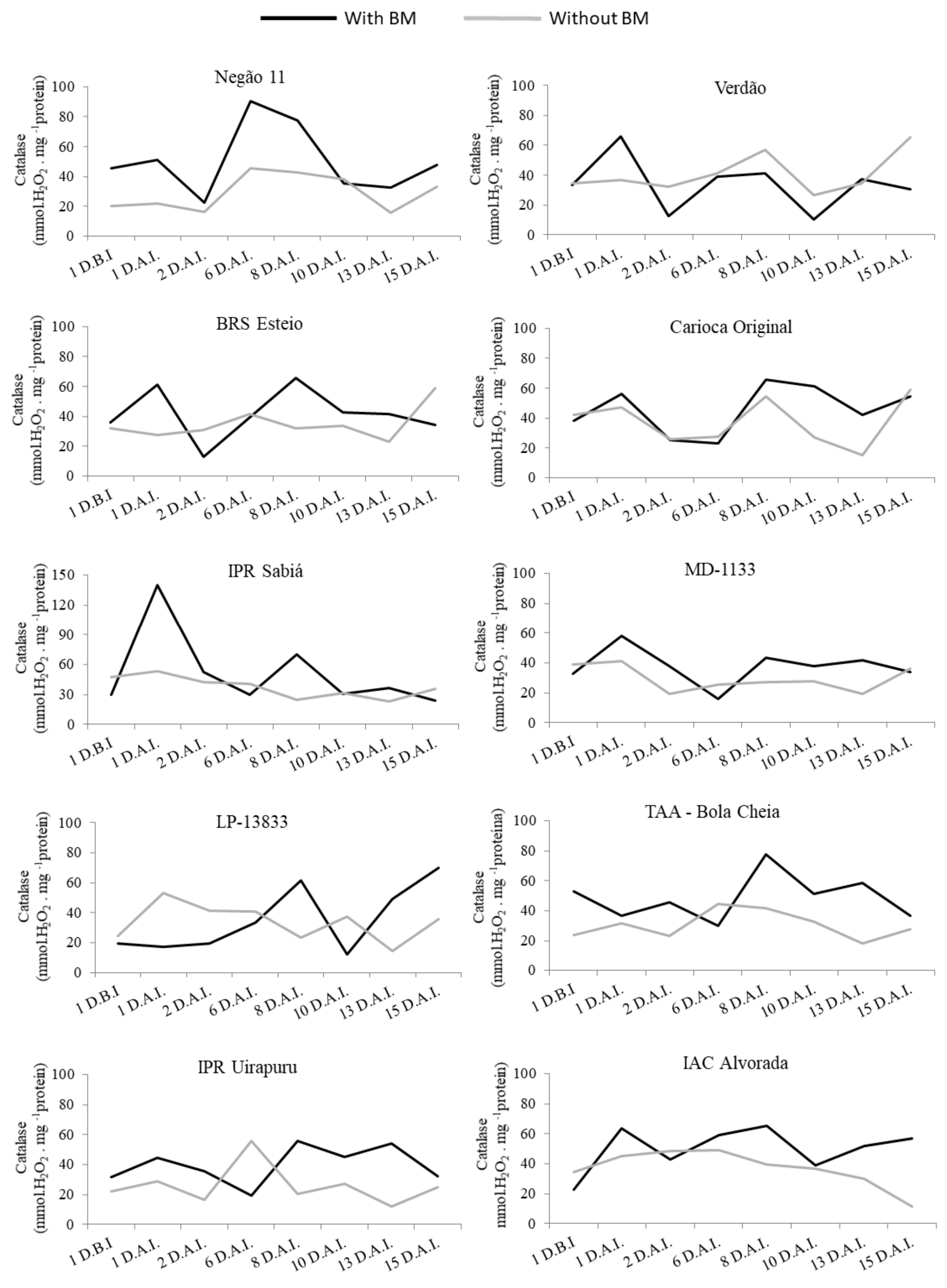

Figure S3: Catalase content in different common bean genotypes with (dark line) and without (grey line) broad mite - BM (*Polyphagotarsonemus latus*) infestation. Numbers in the x-axis refers at days before infestation (D.B.I,) or days after infestation (D.A.I). Londrina-PR, 2020.

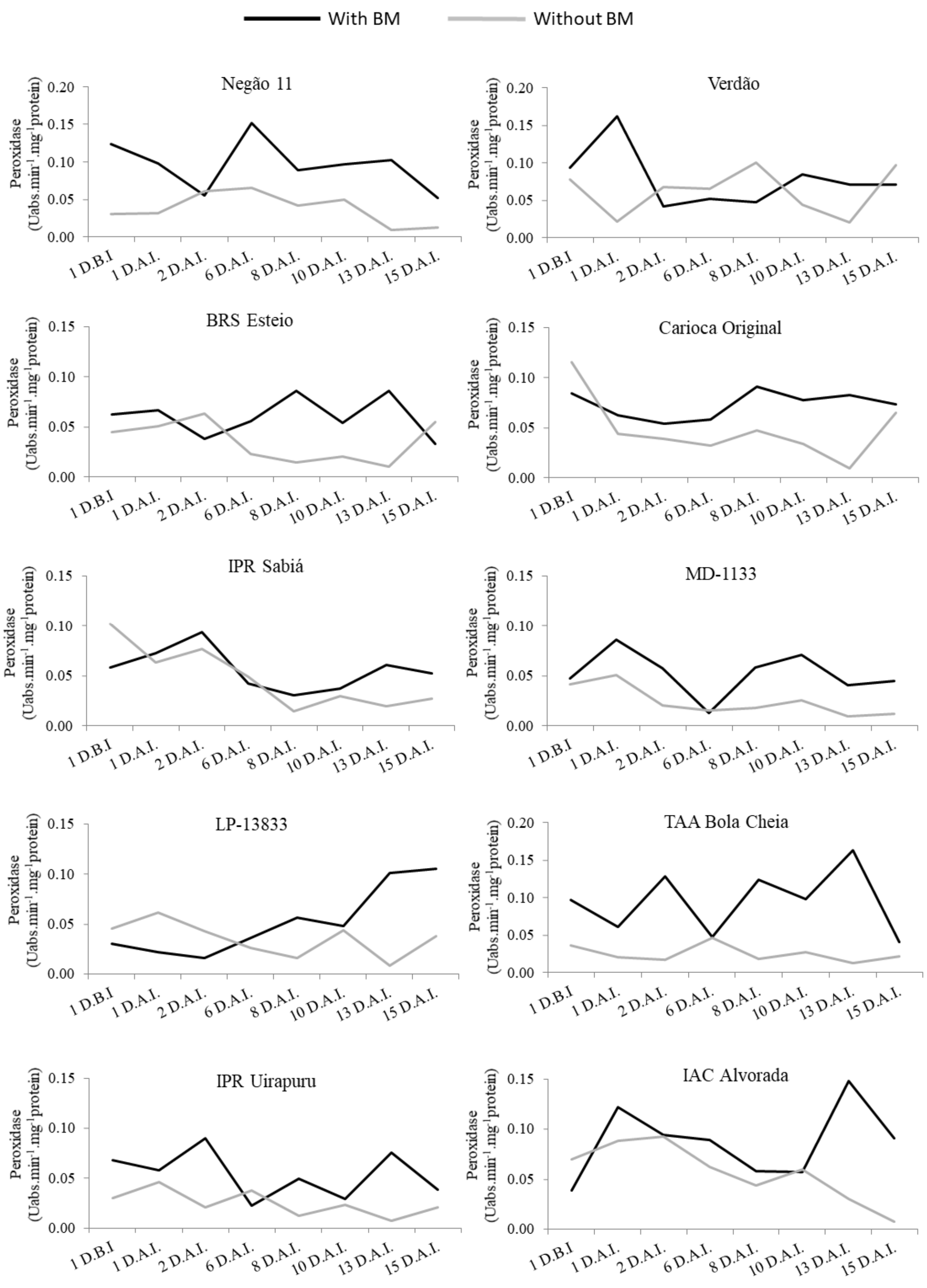

Figure S4: Peroxidase content in different common bean genotypes with (dark line) and without (grey line) broad mite - BM (*Polyphagotarsonemus latus*) infestation. Numbers in the x-axis refers at days before infestation (D.B.I,) or days after infestation (D.A.I). Londrina-PR, 2020.

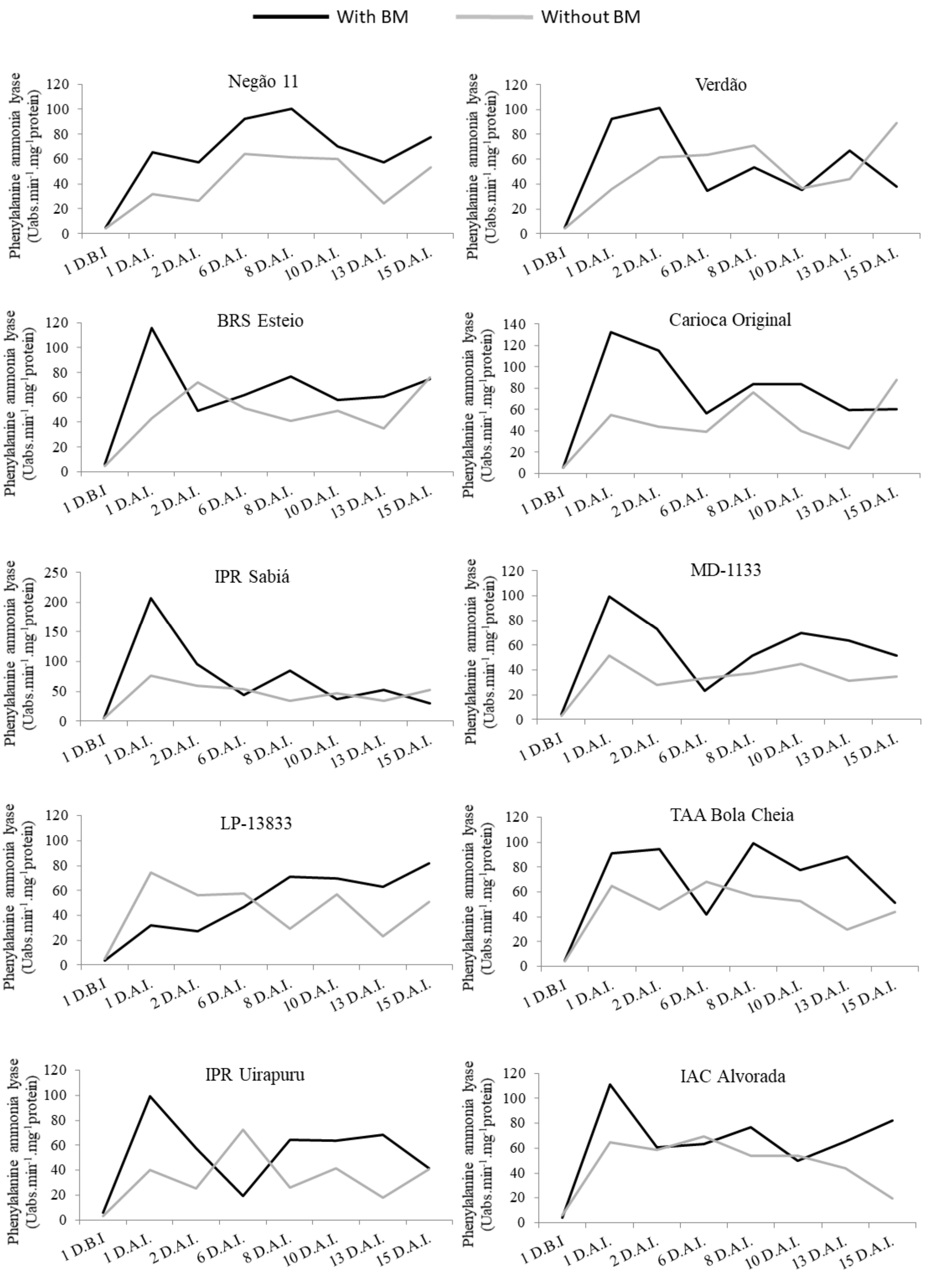

Figure S5: Phenylalanine ammonia lyase content in different common bean genotypes with (dark line) and without (grey line) broad mite - BM (*Polyphagotarsonemus latus*) infestation. Numbers in the x-axis refers at days before infestation (D.B.I,) or days after infestation (D.A.I). Londrina-PR, 2020.
